# Energy Drink-Related Cardiovascular Presentations in Children and Adolescents: A Narrative Review and Practical Guide for Management

**DOI:** 10.7759/cureus.103855

**Published:** 2026-02-18

**Authors:** Eleni Kiose, Zafeiria Titsi, Dimos Mademidis, Dimitrios-Nektarios Iatrou, Dimitrios Kourdakis, Prodromos Bampageorgakas, Antigoni Deri

**Affiliations:** 1 Padiatrics, General Hospital of Komotini, Komotini, GRC; 2 Cardiology, General Hospital of Komotini, Komotini, GRC; 3 Pediatrics, General Hospital of Thessaloniki "G. Gennimatas", Thessaloniki, GRC; 4 Radiology, AHEPA University General Hospital, Thessaloniki, GRC; 5 Pediatric Cardiology, Leeds Teaching Hospitals Trust, Leeds, GBR

**Keywords:** adolescents, arrhythmia, caffeine, cardiovascular effects, children, emergency department, energy drinks, palpitations

## Abstract

Energy drink (ED) consumption has become increasingly common among children and adolescents, raising questions about its possible effects on cardiovascular health. Owing to ongoing physiological development and sensitivity to stimulant ingredients, minors may experience distinct responses to these beverages. This review provides an overview of ED components and discusses their potential influence on cardiovascular function in younger populations, as well as the range of symptoms reported in clinical settings. Attention is given to how stimulant-containing drinks may affect heart rate, rhythm, and overall circulatory responses, particularly with frequent or excessive intake. The review also considers broader effects related to sleep and behavioral regulation. Emphasis is placed on the importance of routinely considering ED use when evaluating pediatric patients with cardiovascular complaints and on the need for increased awareness regarding consumption habits among minors.

## Introduction and background

Over the past two decades, the consumption of energy drinks (EDs) - sweetened, caffeinated beverages containing stimulants such as taurine, guarana, ginseng, and sugars - has risen dramatically among children and adolescents worldwide [1-4]. Vigorous marketing, peer influence, and misconceptions about enhanced performance or alertness have contributed to their growing popularity among juveniles [2,3]. According to the European Food Safety Authority (EFSA), approximately two-thirds of European adolescents aged 10-18 years report ED consumption, with 12% classified as high acute consumers (>1L per session) [1]. Comparable trends have been reported globally: in Korea, the proportion of adolescents consuming EDs three or more times per week increased nearly fourfold between 2014 and 2019 [5], while in Canada, 73.6% of youth aged 12-17 years reported prior ED use, and over half experienced at least one adverse effect, most commonly tachycardia, palpitations, or chest pain [6,7].

Children and adolescents are physiologically more susceptible to the stimulant effects of EDs because of lower body mass, limited caffeine tolerance, and individual variability in caffeine metabolism [8]. The primary active ingredients, particularly caffeine, exert dose-dependent cardiovascular effects, including: a) increased blood pressure, b) altered heart rate, and c) arrhythmogenic potential [9,10]. Controlled pediatric crossover studies have demonstrated that even moderate, body-weight-adjusted ED doses (3 mg caffeine/kg) significantly increase systolic and diastolic blood pressure [11], elevate arterial stiffness [12], decrease left ventricular efficiency [13], and induce supraventricular extrasystoles [10]. These findings highlight the vulnerability of the developing cardiovascular system to stimulant-induced hemodynamic stress.

Epidemiological and toxicological data corroborate these experimental results. Between 2011 and 2023, United States (U.S.) Poison Centers (America’s Poison Centers) recorded more than 32,000 pediatric exposures to caffeine-containing energy products, with a 17% annual rise in reports; adolescents had more than 12 times the likelihood of requiring hospital admission and nearly nineteen times the risk of experiencing serious medical outcomes compared to younger children [14]. Nordt et al. reported that among adolescent emergency department patients, 40% of ED consumers experienced adverse effects, with insomnia (19%), jitteriness (19%), palpitations (16%), and chest pain (5%) being the most prevalent [15]. Likewise, a systematic review of pediatric case reports found that nearly half of all ED-related adverse events in minors involved the cardiovascular system, ranging from palpitations and syncope to supraventricular or ventricular arrhythmias and myocardial ischemia [9,16].

Chronic and high-frequency ED consumption is also associated with clustering of other cardiovascular risk behaviors, including alcohol use, smoking, and sleep deprivation [17-19]. The German Energy Drinks and Cardiological Risk (EDKAR) study identified higher rates of such risk behaviors among adolescents consuming EDs four or more days per week at ≥3 mg caffeine/kg/day [18]. Moreover, co-ingestion of alcohol and EDs has been linked to increased emergency visits, risk-taking, and blunted perception of intoxication [3,19]. Despite these growing safety concerns, uniform global regulations restricting ED sales to minors remain absent, prompting calls for World Health Organization (WHO)-supported legislative frameworks analogous to tobacco control conventions [20].

Given the rising prevalence of ED consumption among youth and the increasing number of emergency presentations for chest pain, palpitations, and other cardiovascular complaints following consumption, a systematic understanding of this phenomenon is essential. This narrative review synthesizes the available evidence on children, teenagers, and adolescents presenting to emergency departments or hospitals with thoracic or chest pain and palpitations after ED consumption, focusing on epidemiological trends, underlying mechanisms, and implications for pediatric clinical care and prevention.

## Review

Materials and methods

This narrative review was conducted to synthesize current evidence on the cardiovascular effects of ED consumption in children and adolescents. A comprehensive literature search was performed in PubMed database using combinations of relevant keywords and Medical Subject Headings (MeSH) where applicable, including EDs, caffeine, children, adolescents, pediatrics, cardiovascular effects, heart rate, blood pressure, arrhythmia, palpitations, chest pain, emergency department, toxicity, and stimulant beverages.

The literature search encompassed studies published between January 2000 and August 2025, capturing the period during which ED consumption became increasingly prevalent and systematically examined in pediatric populations. From each eligible publication, data were extracted where available on the first author, year of publication, country or region of study, study design (including randomized controlled trials, observational studies, case series, case reports, systematic reviews, and poison center or registry analyses), study population characteristics such as age range and sample size, the type and amount of ED or specific constituent exposure, reported cardiovascular outcomes, and key clinical findings.

Studies were included if they met at least one of the following criteria: original research involving children or adolescents (≤18 years) examining cardiovascular or related physiological effects of ED consumption; emergency department, hospital-based, or poison center studies reporting cardiovascular presentations following ED intake in minors; clinical trials assessing hemodynamic or electrophysiological outcomes after ED or caffeine exposure in pediatric populations; or relevant systematic or narrative reviews providing pediatric-specific cardiovascular data and publications available in the English language. Studies were excluded if they focused exclusively on adults (>18 years) without separate pediatric data, addressed sports drinks without stimulant content, were non-English publications, consisted solely of conference abstracts without full-text availability, or lacked cardiovascular or clinically relevant physiological outcomes. Owing to the heterogeneity of study designs, populations, and reported outcomes, a qualitative narrative synthesis approach was adopted rather than a formal meta-analysis.

Epidemiology of ED consumption among minors

The global increase in ED consumption among minors has been accompanied by a parallel rise in emergency department visits and hospital admissions linked to cardiovascular complaints such as palpitations, chest pain, and arrhythmias. Registry-based data and national surveillance systems indicate that this is not an isolated phenomenon but a growing public health concern. According to the U.S. Drug Abuse Warning Network (DAWN), emergency visits related to ED consumption demonstrated a tenfold increase between 2005 and 2009 (from 1,128 to 13,114 cases) and doubled again between 2007 and 2011, reaching 20,783 cases annually; approximately 92% of these presentations were attributed to adverse reactions rather than intentional misuse [16]. More recent data from America’s Poison Centers (2011-2023) documented 32,482 pediatric exposures to caffeine-containing energy products, reflecting a 17% annual increase; teenagers aged 13-19 years accounted for most severe cases, being over twelve times more likely to require hospital admission and nearly nineteen times more likely to experience serious outcomes than younger children. The predominant clinical symptoms reported included palpitations, chest discomfort, tremor, and anxiety, with 15-20% of moderate-to-severe cases requiring cardiovascular evaluation or monitoring [14].

International epidemiological trends mirror these findings. The EFSA multicenter survey of 52,000 participants across 16 European Union (EU) countries revealed that 68% of adolescents (10-18 years) consumed EDs, and 12% were categorized as “high acute” consumers (>1L per session) [21]. National and regional studies confirm similar prevalence rates: 67% of Polish adolescents reported ED use, with 15% experiencing palpitations or overexcitement after consumption [22]; in Canada, 73.6% of youth aged 12-17 years reported having consumed EDs, and more than half experienced adverse effects, most frequently tachycardia, chest pain, and palpitations [6]; in Korea, the proportion of adolescents consuming EDs three or more times weekly increased nearly fourfold between 2014 and 2019 [5].

Emergency medicine research reinforces these epidemiological data. In a U.S. pediatric and adolescent ED cohort, 53% of patients reported ED use within the previous month, and users exhibited significantly higher rates of physiologic adverse effects, including palpitations, restlessness, and chest discomfort, compared with traditional caffeine consumers [16]. Similarly, Nordt et al. documented that 16% of adolescent ED patients reported palpitations and 5% chest pain following ED consumption [15]. Collectively, registry data, poison surveillance, and clinical studies converge on the finding that cardiovascular symptoms - chiefly palpitations and chest pain - are the predominant reason for ED-related emergency visits among children and adolescents.

Composition and mechanisms of action of ED

EDs constitute a heterogeneous group of beverages with wide variability in formulation, caffeine content, and additive profiles. More than 300 commercial brands exist globally, but most share five functional categories of ingredients: a) caffeine, b) sugars or artificial sweeteners, c) amino acids (notably taurine and L-carnitine), d) B-group vitamins, and e) herbal or plant-derived stimulants such as guarana, ginseng, ginkgo biloba, and yerba maté [2,12,15,22]. The caffeine content of a 250mL can ranges from 80mg to more than 200mg, while larger servings may contain up to 500mg - levels that equal or exceed those in a cup of coffee and substantially surpass the pediatric safety threshold of <100mg/day recommended by the American Academy of Pediatrics [23].

Caffeine acts primarily as a nonselective adenosine receptor antagonist, increasing catecholamine release and sympathetic outflow, thereby elevating heart rate and blood pressure [8]. Guarana and kola nut add additional methylxanthines, often augmenting total caffeine load and prolonging stimulant effects [4]. Taurine, frequently included at concentrations up to 4000mg/L, influences intracellular calcium handling, myocardial contractility, and excitation-contraction coupling, with potential synergistic interaction with caffeine to enhance inotropic and chronotropic effects [10]. Glucuronolactone, another common additive, participates in carbohydrate metabolism and purported detoxification pathways, though data on its cardiovascular safety in minors remain limited [22]. B-vitamins serve as metabolic cofactors involved in energy metabolism, whereas herbal additives such as ginseng and ginkgo may exert mild sympathomimetic or vasodilatory actions [3]. The principal components of ED and their cardiovascular mechanisms are summarized in Table 1.

**Table 1:** Composition, mechanism of action and cardiovascular effects of ED ingredients This table is compiled using data from multiple sources [1-4,8,10-12,12-14,18,19,22-27]. AAP: American Academy of Pediatrics; ED: Energy drink; RDA: Recommended dietary allowance, ↑: Increased/Elevation; ↓: Decreased/Reduction

Component	Common Sources/Examples	Typical Concentration	Primary Mechanism of Action	Cardiovascular or Physiologic Effects	Notes/Safety Considerations
Caffeine	Synthetic caffeine, guarana, kola nut	80-500mg per serving	Nonselective adenosine receptor antagonist; increases catecholamine release	↑ Heart rate, ↑ blood pressure, ↑ myocardial workload	Exceeds pediatric safety threshold (<100 mg/day) per AAP
Sugars - Artificial Sweeteners	Sucrose, glucose, sucralose, aspartame	Variable	Provide rapid energy or sweetness	Hyperglycemia, metabolic load	Excess intake linked to obesity, insulin resistance
Amino Acids	Taurine, L-carnitine	Taurine up to 4,000 mg/L	Modulates calcium handling and myocardial contractility	Enhances inotropic and chronotropic effects, especially with caffeine	Potential synergistic cardiac stimulation
B-Group Vitamins	B2, B3, B6, B12	Variable (often 100-500% RDA)	Cofactors in energy metabolism	Support oxidative metabolism	Generally safe, though high doses may cause neuropathy (B6)
Herbal/Plant Stimulants	Guarana, ginseng, ginkgo biloba, yerba maté	Variable	Sympathomimetic and vasodilatory properties	Augments stimulant effects, mild ↑ blood pressure/heart rate	Additive effects with caffeine; unpredictable potency
Glucuronolactone	Synthetic additive	Variable	Involved in carbohydrate metabolism, detoxification	Minimal direct cardiovascular data	Safety in minors not well established

The combination of these stimulants can potentiate myocardial workload and excitability, explaining the recurrent emergency presentations of tachyarrhythmias, palpitations, and chest pain reported in pediatric populations [9,14,15].

Adverse effects associated with ED consumption

The clinical effects of ED consumption encompass cardiovascular, neurological, metabolic, and gastrointestinal systems, with severity dependent on dose, individual susceptibility, and co-ingestion with alcohol or other stimulants. Cardiovascular manifestations are the most frequently reported, including palpitations, chest pain, tachycardia, hypertension, and documented arrhythmias such as supraventricular tachycardia (SVT) or premature ventricular contractions [9,14-16]. Neurological symptoms include agitation, anxiety, tremor, dizziness, headache, and insomnia [16]. Gastrointestinal complaints (nausea, vomiting, abdominal pain) and metabolic disturbances such as hyperglycemia or hypokalemia have also been described [3,11,22].

Severe but rare complications, reported mainly in adolescents and young adults, include seizures, myocardial ischemia, QT prolongation, and, in isolated cases, cardiac arrest [9,16,24]. The American Academy of Pediatrics explicitly advises that children and adolescents should avoid ED consumption due to such potential health risks [23]. The spectrum of adverse effects associated with ED consumption across major organ systems is summarized in Table 2.

**Table 2:** Reported adverse effects associated with ED consumption This table is compiled using data from multiple sources [3,9,11,14-16,22,24]. ED: Energy drink; SVT: Supraventricular tachycardia

System Affected	Reported Effects/Symptoms	Severity Range	Contributing Factors
Cardiovascular	Palpitations, chest pain, tachycardia, hypertension, SVT, premature ventricular contractions	Mild to severe	High caffeine dose, co-ingestion with other stimulants, individual sensitivity
Neurological	Agitation, anxiety, tremor, dizziness, headache, insomnia, seizures (rare)	Mild to severe	Caffeine toxicity, stimulant synergy, genetic susceptibility
Gastrointestinal	Nausea, vomiting, abdominal pain	Mild	Gastric irritation, excess caffeine or sugar
Metabolic	Hyperglycemia, hypokalemia	Mild to moderate	High sugar content, catecholamine surge, dehydration
Severe/Rare Complications	Myocardial ischemia, QT prolongation, cardiac arrest	Severe/Life-threatening	Excessive caffeine, arrhythmogenic predisposition, alcohol co-use

Ingredient-specific associations with reported symptoms

Caffeine is the principal compound implicated in most acute adverse effects. Excessive caffeine intake increases intracellular cyclic adenosine monophosphate (cAMP) and catecholamine levels, leading to tachycardia, elevated blood pressure, palpitations, tremor, anxiety, and insomnia [8]. Taurine, though often described as cardioprotective in moderation, may potentiate caffeine’s chronotropic effects through modulation of calcium flux, predisposing to arrhythmogenic potential when consumed together [10]. Guarana and kola nut, rich in methylxanthines, act synergistically with caffeine to exacerbate sympathomimetic stimulation [4].

High sugar content contributes to metabolic and cardiovascular strain, promoting hyperglycemia and dehydration, which can indirectly trigger tachycardia and fatigue [3,22]. Herbal extracts such as ginseng and ginkgo biloba may enhance sympathetic activity and platelet aggregation or interact with medications, further complicating cardiovascular status in susceptible adolescents [3,25]. In toxicological reports, the majority of cardiovascular and neurological symptoms, including palpitations, hypertension, chest pain, tremor, and anxiety, are linked primarily to caffeine and its synergistic interactions with taurine and guarana rather than to vitamins or glucuronolactone [3,9,14,16]. Ingredient-specific associations between ED constituents and reported symptoms are summarized in Table 3.

**Table 3:** Ingredient-specific associations with reported adverse effects of EDs This table is compiled using data from multiple sources [3,4,8-10,14,16,22,25]. cAMP: Cyclic adenosine monophosphate; ↑: Increased/Elevation; ED: Energy drink

Ingredient/Component	Primary Mechanism of Action	Associated Clinical Effects/Symptoms	Potential Synergistic or Interacting Compounds
Caffeine (Adenosine Receptor Antagonism)	Inhibits phosphodiesterase: ↑ cAMP and catecholamines	Tachycardia, hypertension, palpitations, tremor, anxiety, insomnia	Guarana, kola nut, taurine
Taurine	Modulates intracellular Ca²⁺ flux and myocardial contractility	May potentiate caffeine’s chronotropic and inotropic effects; arrhythmogenic potential at high doses	Caffeine
Guarana/Kola Nut	Source of methylxanthines (‘’additional caffeine’’, theobromine)	Augmented sympathetic stimulation, insomnia, tremor, palpitations	Caffeine, taurine
Sugars/Carbohydrates	Rapid glucose absorption: transient hyperglycemia and osmotic diuresis	Hyperglycemia, dehydration, tachycardia, fatigue	-
Herbal Extracts (Ginseng, Ginkgo Biloba)	Mild sympathomimetic and platelet-aggregating properties; possible drug interactions	↑ Blood pressure, nervousness, potential bleeding risk	Caffeine, cardiovascular medications
Vitamins/Glucuronolactone	Metabolic cofactors and detoxification roles	Generally minimal direct cardiovascular or neurologic impact	-

Quantities and doses associated with adverse effects

The dose required to elicit adverse effects from ED constituents differs among individuals based on age, body mass, and caffeine tolerance. In pediatric populations, randomized controlled trials demonstrate significant increases in systolic and diastolic blood pressure and supraventricular ectopy following ED doses equivalent to approximately 3mg caffeine/kg body weight, corresponding to one standard 250mL can for a 40-50 kg adolescent [10,11,13]. These effects are clearly documented in controlled pediatric trials [10-13] (Table 4). However, discrepancies between labeled and measured caffeine content have been reported in some commercial products, potentially complicating accurate estimation of real-world exposure. Data on sex- or pubertal-stage-specific susceptibility remain limited.

**Table 4:** Evidence for severe and life-threatening cardiovascular outcomes associated with ED consumption in children and adolescents This table is compiled using data from multiple sources [1,3,9-16,24,25]. ED: Energy drink; ECG: Electrocardiogram; ↑: Increased/Elevation; ↓: Decreased

Evidence Type	Study	Population/Setting	Exposure Context	Cardiovascular Outcomes Reported	Life-Threatening Outcomes	Key Notes on Certainty
Randomized Controlled Trials	Mandilaras et al. [10]	Healthy children & teenagers	~3 mg caffeine/kg	ECG interval changes, supraventricular extrasystoles	No	Assess physiologic and electrophysiological changes; NOT powered for rare severe events
	Oberhoffer et al. [1]	Healthy children & teenagers	Acute ED exposure	↑ Heart rate and blood pressure	No	Demonstrates acute hemodynamic stress only
	Oberhoffer et al. [11]	Healthy children & teenagers	Acute ED exposure	↑ Ambulatory blood pressure	No	No malignant arrhythmias observed
	Li et al. [12]	Healthy children & teenagers	Acute ED exposure	↑ Arterial stiffness	No	Subclinical vascular effects
	Oberhoffer et al. [13]	Healthy children & teenagers	Acute ED exposure	↓ Left ventricular efficiency	No	Functional changes without clinical events
Emergency Department Studies	Nordt et al. [15]	Adolescent ED patients	Recent ED intake	Palpitations, chest pain, tachycardia	Rare/severe events not primary focus	Symptom-driven cohort
	Jackson et al. [16]	Adolescent & young adult ED patients	ED and caffeine use	Arrhythmias, chest pain, neurologic symptoms	Yes (rare)	Severe outcomes reported but uncommon
Poison Center/Registry Data	Thompson et al. [14]	America's Poison Centers (children/adolescents)	Caffeine-containing energy products	Tachyarrhythmias, seizures, severe toxicity	Yes	Dose-dependent risk; higher severity in adolescents
Systematic/Narrative Reviews	Li et al. [9]	Pediatric literature review	ED exposure	Palpitations, QT prolongation, ischemia	Yes	Life-threatening events rare; based on case reports
	De Sanctis et al. [3]	Adolescents	ED consumption	Arrhythmias, ischemic events	Yes	Predominantly observational and case-based
Toxicology Reviews	Nawrot et al. [24]	All ages	High-dose caffeine	Ventricular arrhythmias, cardiac arrest	Yes	Lethal doses (~5-10g) described, mainly adults
Case Reports/Series	Reissig et al. [25]	Adolescents & young adults	Excessive ED intake	QT prolongation, arrhythmias	Yes	Attribution limited by co-ingestants and dose uncertainty

In adults, symptomatic responses such as jitteriness and palpitations typically occur at caffeine doses >400mg/day or following consumption of >500mL of EDs containing >150-200mg caffeine per serving [8,24]. Toxicity, including arrhythmia or seizures, has been reported with total caffeine intake exceeding 10mg/kg in children and >1g/day in adults [9,14,16,24]. Lethal outcomes in adults have been reported with massive caffeine ingestion, typically at doses of approximately 10g or higher [24]. Taurine is generally well tolerated up to 3g/day, but co-ingestion with caffeine may lower the threshold for cardiovascular stimulation [10]. Sugar content in commercial EDs ranges from 30-60g per 500mL, exceeding daily pediatric recommendations and contributing to transient tachycardia, dehydration, and hyperglycemia [3,22,23].

These findings confirm that even moderate ED consumption within widely available commercial serving sizes can elicit measurable cardiovascular effects in children and adolescents, emphasizing the absence of a clear “safe” dose threshold in this age group. Quantities and doses of ED constituents associated with adverse effects across age groups are summarized in Table 5.

**Table 5:** Quantities and doses of ED constituents associated with adverse effects This table is compiled using data from multiple sources [3,8-14,16,22-24]. ED: Energy Drink, ↑: Increased/Elevation

Constituent	Approximate Dose/Concentration	Population/Context	Observed Effects
Caffeine	~3 mg/kg body weight (≈250mL ED for 40-50 kg adolescent)	Children/Adolescents	↑ Systolic & diastolic blood pressure, supraventricular ectopy
Caffeine	>400mg/day or >500mL ED (150-200mg caffeine per serving)	Adults	Jitteriness, palpitations, anxiety
Caffeine (Toxic Range)	>10mg/kg (children), >1 g/day (adults)	Children & adults	Arrhythmia, seizures, cardiac arrest
Taurine	≤3g/day (typical intake)	General population	Generally well tolerated; may potentiate caffeine’s cardiovascular effects
Sugars	30-60g per 500mL serving	Children/Adolescents	Hyperglycemia, dehydration, tachycardia, fatigue

Short-term and long-term adverse effects of regular ED consumption

Short-Term Effects

Acute consumption of EDs can precipitate a spectrum of transient but clinically relevant effects across multiple organ systems. Cardiovascular symptoms such as palpitations, tachycardia, transient hypertension, and chest pain are the most commonly reported in both pediatric and adult studies [9,14-16]. Neurological manifestations include anxiety, jitteriness, tremor, insomnia, and in severe cases, seizures or agitation secondary to acute caffeine intoxication [16,24]. Gastrointestinal discomfort (nausea, vomiting, abdominal pain) and metabolic responses such as hyperglycemia or dehydration are frequent in adolescents who consume large quantities rapidly or concomitantly with exercise [3,22]. In controlled pediatric crossover trials, even single doses of 3mg caffeine/kg body weight induced measurable rises in blood pressure and supraventricular ectopy, indicating that “acute” exposure levels commonly achievable through commercial EDs can provoke cardiovascular stress in minors [10-13].

Long-Term Effects

Frequent consumption of EDs by adolescents is increasingly viewed as a potential behavioral and physiological hazard to health. Regular consumption has been associated with sustained elevations in resting blood pressure, sleep disruption, and increased sympathetic tone, potentially predisposing to early vascular dysfunction and endothelial impairment [18,19,26]. Repeated exposure to high-caffeine, high-sugar formulations may alter glucose metabolism, insulin sensitivity, and body weight regulation, promoting metabolic syndrome components [3,22,26]. Evidence from the EDKAR and additional adolescent cohorts indicates that behavioral effects encompass tolerance, dependence, and risk-taking behaviors involving the combined use of alcohol, tobacco, and illicit drugs [17-19]. Sleep deprivation and fatigue cycles reinforced by chronic stimulant intake further compound cardiovascular strain and cognitive dysfunction [18,19].

Despite the limited availability of long-term controlled pediatric research, accumulating observational evidence indicates that frequent ED intake in adolescence is linked to increased systolic blood pressure, diminished heart rate variability, and decreased sleep duration, all of which are recognized predictors of cardiovascular disease risk [26,27]. These findings underscore the potential for ED consumption to contribute not only to acute emergency presentations but also to chronic cardiovascular risk trajectories extending into adulthood.

The short-term as well as the long-term adverse effects associated with regular ED consumption are summarized in Table 6.

**Table 6:** Short-term and long-term adverse effects of regular ED consumption This table is compiled using data from multiple sources [3,9-19,22,24-27]. ↑: Increased/Elevation; ED: Energy drink

System/Domain	Short-Term Effects	Long-Term Effects	Potential Mechanisms/Contributing Factors
Cardiovascular	Palpitations, tachycardia, transient hypertension, chest pain, supraventricular ectopy	Sustained ↑ resting blood pressure, reduced heart rate variability, endothelial dysfunction, early vascular changes	Sympathetic overactivation, elevated catecholamines, chronic caffeine exposure
Neurological	Anxiety, jitteriness, tremor, insomnia, agitation, seizures (in toxicity)	Sleep disruption, dependence, cognitive fatigue, altered stress response	Caffeine-induced arousal, sleep cycle disturbance, tolerance development
Metabolic	Hyperglycemia, dehydration, transient fatigue	Altered glucose metabolism, insulin resistance, weight dysregulation, metabolic syndrome	Excess sugar intake, repeated sympathetic stimulation
Gastrointestinal	Nausea, vomiting, abdominal pain	Possible gastritis or gastrointestinal irritation with chronic use	Caffeine and sugar-induced gastric acid secretion
Behavioral/Psychosocial	Increased alertness, agitation	Tolerance, dependence, co-use with alcohol, tobacco, and illicit drugs, risk-taking behaviors	Dopaminergic reinforcement, peer and psychosocial influences
Sleep/Cognitive	Insomnia, reduced sleep duration	Chronic sleep deprivation, impaired cognitive performance	Recurrent stimulant intake, disruption of circadian rhythm

Emergency department red flags, differential diagnosis, and management

Red-Flag Presentations

In the emergency department, certain symptoms and vital-sign abnormalities following ED intake warrant urgent evaluation. Thresholds should be interpreted using age-adjusted pediatric norms. Red flags include: (a) Persistent or severe chest pain, particularly with radiation or associated diaphoresis; (b) Palpitations accompanied by dizziness, syncope, or near-syncope; (c) Sustained tachycardia (>120bpm) or new-onset arrhythmias on electrocardiogram; (d) Hypertension refractory to rest or associated with neurological symptoms (e.g., headache, confusion); (e) Seizures, agitation, or altered mental status suggestive of severe caffeine toxicity; and (f) Evidence of myocardial ischemia (ST-segment changes, elevated troponin) [9,14-16,24]. Adolescents presenting with these features should be triaged as high priority and undergo continuous cardiac and hemodynamic monitoring.

Differential Diagnosis

The differential diagnosis for thoracic or chest pain and palpitations in minors following ED consumption includes: (a) Primary caffeine toxicity or sympathomimetic excess (most common); (b) SVT, premature atrial/ventricular contractions, or long-QT-related arrhythmias; (c) Myocarditis or pericarditis unrelated to ED intake (viral or post-infectious); (d) Anxiety/panic attacks, hyperventilation, or dehydration-induced tachycardia; (e) Coronary vasospasm or rare cases of myocardial infarction secondary to high caffeine/taurine load; and (f) Electrolyte disturbances (e.g., hypokalemia) contributing to arrhythmogenesis [3,9,10,22,24]. Baseline cardiac history should lower the threshold for investigation.

Emergency Management: A Conceptual Framework for Clinical Protocols

Management should be primarily symptom-directed and supportive, beginning with prompt initial stabilization, including assessment of the airway, breathing, and circulation, along with continuous cardiac monitoring. An electrocardiogram (ECG) and evaluation of serum electrolytes - particularly potassium, magnesium, and calcium - should be performed to identify potential arrhythmias or electrolyte disturbances. Liver, renal, and thyroid function tests are recommended to exclude underlying metabolic or endocrine contributors to tachyarrhythmia or autonomic symptoms. Intravenous fluid therapy should be initiated to correct dehydration and enhance caffeine clearance. Benzodiazepines, such as diazepam or lorazepam, may be administered to manage agitation, seizures, or severe tremors associated with caffeine toxicity. In cases of severe tachyarrhythmias that are unresponsive to conservative measures, β-blockers (e.g., propranolol) may be used cautiously, provided there is no evidence of hypotension or heart block. Activated charcoal may be considered if the patient presents within one hour of ingestion. Consultation with pediatric cardiology or toxicology is advised for patients with significant arrhythmias, syncope, or elevated troponin levels. Given the prolonged half-life of caffeine in adolescents - up to 10 hours - and the potential for delayed arrhythmia, observation for at least six-eight hours is recommended in symptomatic minors. Long-term management should include counseling on stimulant avoidance, adequate hydration, and good sleep hygiene [24,25].

Future directions

Despite growing recognition of the cardiovascular effects associated with ED consumption in children and adolescents, several important knowledge gaps remain. Future research should prioritize well-designed, pediatric-specific studies to better define safe intake thresholds for caffeine and other commonly used ED constituents, taking into account age, body mass, sex, and interindividual variability in stimulant metabolism. Longitudinal cohort studies are particularly needed to clarify the potential long-term cardiovascular, metabolic, and autonomic consequences of repeated ED exposure during critical developmental periods. Notably, current conclusions regarding chronic exposure are derived primarily from longitudinal and cross-sectional observational data, as randomized controlled trials in pediatric populations are lacking. 

Further investigation into the combined and potentially synergistic effects of ED ingredients such as caffeine, taurine, sugars, and herbal stimulants, on myocardial electrophysiology, vascular function, and blood pressure regulation is warranted, as most existing studies focus on single compounds rather than real-world formulations. Research addressing vulnerable subgroups, including adolescents with underlying cardiac conditions, hypertension, sleep disorders, or concurrent stimulant or alcohol use, would also provide clinically actionable insights.

From a clinical and public health perspective, the development of standardized screening tools for ED consumption in pediatric and adolescent healthcare settings may improve early identification of at-risk individuals. Additionally, establishing coordinated surveillance systems or registries to systematically capture ED-related emergency department visits and hospitalizations could help quantify the true clinical burden and monitor trends over time. Finally, interdisciplinary research integrating clinical data, behavioral science, and policy analysis may inform evidence-based educational strategies and regulatory approaches aimed at reducing preventable cardiovascular risks associated with ED use among minors.

## Conclusions

ED consumption among children and adolescents is increasingly recognized as a potential contributor to cardiovascular concerns in this age group. Even moderate exposure to EDs may affect cardiac and vascular function, particularly in individuals demonstrating increased susceptibility to stimulant effects. The increasing frequency of pediatric presentations involving palpitations or related cardiovascular symptoms following ED intake highlights the necessity for early recognition and systematic clinical assessment. Current evidence is strongest for acute cardiovascular effects, while long-term risks remain less well defined. Healthcare professionals should incorporate a detailed history of ED consumption into the evaluation of minors with unexplained cardiovascular or autonomic manifestations. Preventive efforts should emphasize education on stimulant composition, discourage concomitant alcohol use, and promote moderation of intake to reduce avoidable cardiovascular risks.
